# Supplementary material for: Learning and teaching biological data science in the Bioconductor community
Source: PLoS Comput Biol. 2025 Apr 22;21(4):e1012925. doi: 10.1371/journal.pcbi.1012925 (PMC12013867; doi:10.1371/journal.pcbi.1012925)
Supplement: S1 Table — (PDF) [file pcbi.1012925.s001.pdf]

S1 Table. URLs for Resources Mentioned in the Manuscript

Introduction	
Bioconductor Website	https://www.bioconductor.org
Bioconductor Training Committee	https://training.bioconductor.org
Prerequisites	
The Carpentries	https://carpentries.org
R for Data Science	https://r4ds.hadley.nz
swirl: Learn R, in R	https://swirlstats.com
Data Science Specialization	https://www.coursera.org/specializations/jhu-data-science
Data Analysis for the Life Sciences	https://www.edx.org/xseries/data-analysis-life-sciences
Acquire the Fundamentals	
Courses & Conferences	https://bioconductor.org/help/course-materials
Biological Data Science	https://csama2024.bioconductor.eu
Statistical Analysis of Genome Scale Data	https://meetings.cshl.edu/courses.aspx?course=C-DATA
Yes for CURE	https://vjcitn.github.io/YESCDS/
Analyze Your Data	
YouTube Channel	https://www.youtube.com/user/bioconductor
Workflows	https://www.bioconductor.org/packages/release/workflows
Books	https://bioconductor.org/help/bioconductor-books
Single-Cell Analysis	https://bioconductor.org/books/release/OSCA
Spatial Transcriptomics Analysis	https://lmweber.org/BestPracticesST
Hi-C Analysis	https://bioconductor.org/books/release/OHCA
Microbiome Analysis	https://microbiome.github.io/OMA
Modern Statistics for Modern Biology	https://www.huber.embl.de/msmb/
Connect with the Community	
Support Forum	https://support.bioconductor.org
Developer Mailing List	https://stat.ethz.ch/mailman/listinfo/bioc-devel
Slack Workspace	https://slack.bioconductor.org
Develop a Package	
Contributor Guide	https://contributions.bioconductor.org
Developer Mentorship Program	https://www.bioconductor.org/developers/new-developer-program
Advanced R	https://adv-r.hadley.nz/
R Packages	https://r-pkgs.org/
Carpentries Global Instructor Training Program	
Announcement	https://blog.bioconductor.org/posts/2022-07-12-carpentries-membership

Update on Year 1 Applicants	https://blog.bioconductor.org/posts/2023-02-24-carpentries-update
Update after Year 2	https://blog.bioconductor.org/posts/2025-02-28-carpentries-update/
Instructors and Workshops	https://training.bioconductor.org/carpentry
Bioconductor Carpentry Curricula	
The Carpentries Incubator	https://carpentries-incubator.org
Introduction to Data Analysis with R and Bioconductor	https://carpentries-incubator.github.io/bioc-intro
The Bioconductor Project	https://carpentries-incubator.github.io/bioc-project
RNA-seq Analysis with Bioconductor	https://carpentries-incubator.github.io/bioc-rnaseq
Orchestrating Large-Scale Single-Cell Analysis With Bioconductor	https://carpentries-incubator.github.io/bioc-scrnaseq
Computational Infrastructure	
BioC Workshop Template	https://github.com/Bioconductor/BuildABiocWorkshop
Translations	
Code of Conduct Example	https://bioconductor.github.io/bioc_coc_multilingual
Crowdin Projects	https://bioconductor.crowdin.com
